# Effects of Ethanol Exposure during Lactation on Ultrasonic Vocalizations of Rat Pups upon Their Isolation: Increase in Pup Distress Calls †

**DOI:** 10.3390/brainsci11091249

**Published:** 2021-09-21

**Authors:** Mohd. Ashik Shahrier, Hiromi Wada

**Affiliations:** 1 Department of Psychology, Faculty of Biological Sciences, Rajshahi University, Rajshahi 6205, Bangladesh; 2 Department of Psychology, Faculty of Humanities and Human Sciences, Hokkaido University, Kita 10, Nishi 7, Kita-Ku, Sapporo 060-0810, Japan; wada@let.hokudai.ac.jp

**Keywords:** dam–pup interaction, ethanol, pup isolation, rat pup, ultrasonic vocalization

## Abstract

Recording ultrasonic vocalizations (USVs) is a highly sensitive tool to study the dam–pup social relationships, and USV recordings have been used to study the effects of ethanol on pups. Gestational effects of ethanol on the emission of USVs in rat pups have been studied in our previous research. In the present study, the effects of ethanol given to dams during lactation on the acoustic parameters of USVs emitted by isolated pups were examined. Ethanol was administered to dams from postnatal days (PNDs) 5–21. From PNDs 11–21, the high- and low-ethanol-treated dams were exposed to ethanol-containing water (*v*/*v*) at concentrations of 30% and 15%, respectively. Tap water without ethanol (0%) was provided to the control dams. The pups in all three ethanol-treated groups were separated from the dam and littermates on PNDs 4, 8, 12, and 16, and USVs produced by the pups were recorded for 5 min. It was found that elevated distress USVs with longer duration and higher percentage of frequency modulations were displayed by the pups from the high-ethanol dams. Alterations in USVs were particularly evident in the pups with a reduced body weight at PND 12. This effect might be because high-ethanol dams showed significantly lower intake of higher ethanol-containing water, and consequently, produced lower amount of milk, as well as exhibited poor maternal care. Insufficient maternal care and malnutrition resulted in pup growth retardation and increased mortality rate in the high-ethanol group, which were not observed in the low-ethanol or control pups. Accordingly, the pups in the high-ethanol group experienced elevated negative emotionality during isolation from their dam and increased emission of USVs. Longer duration and increased frequency modulation of pup USVs are expected to be noticed by the dam and to initiate/increase proper maternal care. It is concluded that ethanol given to lactating mothers has more serious consequences on pup development than the gestational ethanol exposure, and has more harmful effects on pups.

## 1. Introduction

A popular earlier notion suggested that due to the lactogenic properties alcoholic beverages facilitate milk let-down and rectify milk insufficiency in lactating women [1,2]. Recently, this notion has changed [3,4], though confusion remains as to its positive or negative effects [5,6]. Alcohol consumed by lactating mothers diffuses into the breast milk, and the sensory qualities of milk in intoxicated mothers are changed during breastfeeding [2]. Altered sensory properties of breast milk result in altered sucking patterns reflected through longer nipple attachment in infants. Consequently, 20% less consumption of breast milk in infants during the 3 to 4 h following their mothers’ consumption of alcoholic beverages was observed [7,8]. Moreover, maternal ethanol exposure in the nursing context was found to disrupt the sleep–wake patterns [9,10] and psychomotor development of infants [11]. Alcohol exposure through breast milk also caused dose-dependent reductions in cognitive abilities of children at ages 6 to 7 years [12].

However, the consequences of lactational alcohol exposure in human infants cannot be tested experimentally due to obvious ethical reasons. In human studies, alcohol consumption during lactation, its availability in breast milk, and infant growth are highly comorbid with the ingestion of substances such as tobacco, marijuana, or other psychoactive drugs and with factors such as nutritional status, drinking during pregnancy, prenatal care, socio-economic status of the family, and genetic variations, which cannot be controlled outside of clinical studies. Animal model studies, therefore, play an important role in examining the effects of lactational alcohol exposure on mother–infant interactions, allowing control of confounding factors such as genetic variations, nutritional status, and multidrug use, as well as timing, dose, duration, and pattern of exposure.

In animal model studies, the early postnatal period (PNDs 4–12) in rats was characterized by a brain growth spurt [13], and high doses of ethanol exposure during this period caused the cell death of specific neuronal populations and resulted in neurobehavioral disorders [14,15]. During this critical period, rat pups whose dams were exposed to ethanol for at least 4 days required more time to attach to the nipple [16] and consumed decreased amount of milk [17]. Notably, ethanol administration from lactation days 5–12 caused growth deficits [18] and a high dose exposure (3 g/kg/day) during the early postnatal period resulted in altered suckling behavior, motor impairments and decreased perioral responsiveness in rat pups [19]. Pups can encode the ethanol-related memories of negative emotional valence while interacting with ethanol intoxicated dam. This was evidenced by experiments showing that pups of ethanol-intoxicated dam associated ethanol odor with novel textures and were found to avoid these textures consistently in locational preference tactile tests [20]. In addition, ethanol exposure during lactation promoted hyperactivity and impaired the gait patterns in pups [21,22].

In dam–pup social interactions, ultrasonic vocalizations (USVs) produced by rat pups play an adaptive role in activating maternal responses towards the endangered pups. In rats, maternal attachment is critical in regulating pups’ physiological homeostasis. Disruptions of the dam–pup relationships resulted in alterations of the pup’s heart rate, circadian rhythm, temperature, and so on [23,24]. Consequently, neonatal rat pups emit USVs in the range of 30 to 65 kHz with an approximate average frequency of 40 kHz [25,26,27] that are directed to their dam and were termed distress calls. Distress USVs produced by pups promote search and retrieval behaviors in the dam and elicit pup-directed behaviors such as pup grooming, suppressing, or decreasing maternal biting and cannibalism. Thus, distress USVs have biological significance in triggering appropriate maternal responses, and thus, increase the chances of pup survival [28,29].

Exposure to early life stressors has been found to change the isolation-induced USVs in pups. Environmental temperature during the first neonatal week [27], and a variety of teratogens [30,31] and other social cues [32] during the second neonatal week play a key role in the emission of isolation-induced USVs in pups. Familiar odors, tactile stimulation, the presence of the dam, and littermates decreased the emission of USVs indicating reduced anxiety [33,34]. Conversely, stressful experimental situations increased the emission of USVs reflecting elevations of anxiety [35,36]. Exposure to prenatal insults such as prenatal stress [37], nitrogen [38], electromagnetic fields [39], and methylmercury [40] caused alterations in the acoustic parameters of isolation-induced USVs in pups. Ethanol as an acute stressor has been found to modulate the distress USVs in pups during development. Prenatal ethanol exposure has been reported to decrease [41] or increase [42] the isolation-induced USVs in pups with no alterations reported in one earlier research [43]. In our recent study [44], ethanol exposure (30%, *v*/*v*) from gestational days 8–20 elevated the number, frequencies, and amplitudes of USVs reflecting more negative emotionality in high-ethanol pups compared to their low-ethanol (15%, *v*/*v*) and control (0%) counterparts. Lactational ethanol exposure, on the other hand, disrupted the typical responses to isolation that were reflected through increased latency to vocalize and decreased number of USVs in pups [30,45]. Contrary to this, after interaction with an ethanol-intoxicated dam during lactation, rat pups displayed high levels of isolation-induced USVs indicating heightened behavioral distress [20]. Thus, it is evident that the isolation-induced USVs in pups were altered following exposure to ethanol during the critical developmental period.

Maternal care such as licking and grooming [46] are induced by USV-emitting pups and lactational ethanol exposure has been found to cause serious modifications in these behaviors as well. Administration of ethanol into the drinking water during lactation impaired the mammary gland development in dams [18,47] and resulted in significant changes in maternal nutritional status and milk production, initiating impaired litter growth [48]. Similar to these results, a study [49] with maternal ethanol consumption (5 g/kg/day) during lactation (PNDs 4–10) resulted in impaired maternal behavior that was reflected in longer latency to retrieve the pups. Furthermore, exposure to ethanol reduced the rectal temperature in dams and caused malnourishment of pups as reflected through the incapability to facilitate maternal attachment [50]. The presence of ethanol as a novel chemosensory cue in milk [51] and/or the excretion of ethanol through respiration, urine, salivation, and perspiration [52] influenced the infantile perception of ethanol-induced physiological and behavioral changes in the dam and altered the social interactions between the dam and the pup [20,53].

The acoustic parameters of isolation-induced USVs in pups differed significantly in number, duration, frequency, amplitude, frequency modulations, and bandwidth [54]. The USV repetition rate ranges between 80 and 90 USVs/min, and the call duration varies between 80 and 150 ms [40,55,56]. Therefore, all acoustic parameters facilitating sound localization by the dam are present in the pups’ isolation calls. The expression of distressful states in rat pups through the emission of USV has been more frequently studied by recording and measuring the duration, frequency, and amplitude of calls along with the number of USVs. Duration, frequency, and amplitude of USVs are crucial acoustic parameters for dam–pup social interactions and these parameters are considered as signals for the dams to identify each pup [57]. However, very few studies have considered acoustic parameters in research to clarify the effects of lactational ethanol exposure on dam–pup social interactions. In this study, which is a continuation of our previous studies [44,58], we examine the number, duration, frequency, amplitude, and frequency modulated USVs in pups exposed to ethanol via breast milk, and to ethanol-altered dam behavior, and predict that ethanol exposure during lactation will change the acoustic parameters of distress USVs of pups upon their isolation.

## 2. Materials and methods

### 2.1. Animals

Wistar-derived pregnant rats were purchased from Japan SLC Inc. (Hamamatsu, Japan) at gestational day (GD) 13. They were housed in standard maternity cages lined with wood shavings. On GD 13, rats were assigned to the high-ethanol, low-ethanol, and control groups based on the doses of ethanol administered to the six dams in each group. They had ad libitum access to rat chow MF (Oriental Yeast Ltd., Sapporo, Japan) and tap water before and after the ethanol-exposure period.

The day of parturition was considered as postnatal day (PND) 0. The total number of pups born from the high-ethanol, low-ethanol and control dams were 59, 65, and 57, respectively. On PND 4, litters were culled to eight pups (four males and four females) per dam to ensure that the experimental conditions in all three groups were standardized. Accordingly, the number of pups was 48 in each of the three groups.

Ethanol exposure period in dams ranged from PNDs 5 to 21 and the experimental timeline for the recording of USVs in pups covered PNDs between 4 and 16. The high- and low-ethanol dams were treated with ethanol (purity = 99.5%; Kanto Chemical Co., Inc., Tokyo, Japan) in tap water along with free access to standard diet (rat chow) throughout the exposure period. On PNDs 5–7, 10% and 5%, on PNDs 8–10, 20% and 10% and on PNDs 11–21, 30% and 15% ethanol (*v*/*v*) in tap water were administered to the high- and low-ethanol dams, respectively. Thus, dams from both groups had ad libitum access to ethanol mixture as the only source of drinking between PNDs 5 and 21 and their pups were exposed to ethanol via the breast milk of their dam. The control dams were exposed to pure tap water throughout the exposure period. Ethanol mixture was prepared in a 200 mL drinking bottle and each bottle was provided to each of the high- and low-ethanol dams. The same content of pure tap water without ethanol was provided to the control dams. Ethanol mixture and the tap water for dams in all three groups were refreshed every day from PNDs 5 to 21. The pattern, doses and days of ethanol administration were adopted from relevant previous studies [18,44,47,58,59,60,61,62,63].

The holding room for the dams and pups was maintained under controlled temperature (22 ± 2 °C), humidity (50% ± 10%), and artificial 12 h light/dark (light: 20:00–08:00 and dark: 08:00–20:00) conditions. The research design was approved by the animal ethics committee of Hokkaido University, and rats used in this study were maintained and treated in accordance with the guidelines for Care and Use of Laboratory Animals, Hokkaido University.

### 2.2. Apparatus

The Sonotrack system (version 2.4.0; Metris, Hoofddorp, The Netherlands) was employed to digitally record and analyze the USVs of rat pups. The Sonotrack system was installed on a personal computer and linked with an ultrasonic microphone affixed in a sound-proof chamber.

### 2.3. USV Recording

A total of 30 pups was used for the USV recording in the present study. USVs were recorded from two pups (one male and one female) randomly selected from the litters of each dam. Thus, 10 pups from five dams in each of the three ethanol groups were used for the USV recording procedure. The pups were repeatedly tested for USV recording on PNDs 4, 8, 12, and 16. Each pup was individually isolated from the dam and littermates in the holding room and placed in a translucent cup (13 cm bottom diameter, 15 cm top diameter, and 15 cm height) for transfer to the experiment room for USV recording. The pup was left alone in the sound-proof dark box. The first 5 min was the period of habituation followed by another 5 min of USV recording. The ultrasonic microphone was positioned at a height of 20 cm from the bottom of the translucent cup. After recording, the body weight of the pup was measured and then it was returned to the dam and littermates. Thus, the pup was isolated from the dam and littermates for 10 min on each day of the recording.

USVs were derived during the dark period of the light–dark cycle with maintained temperature (17–21 °C) and humidity (45–67%) conditions. The other pups were used for play fighting studies in juvenile period in another project.

### 2.4. Statistical Analyses

The automatic mode setting of the Sonotrack system selected and analyzed the recorded USVs produced by the pups. The resolution time was 1 ms, and to reduce background noise, low and high cut-off frequencies for analyses were set to 30 and 90 kHz, respectively. The Sonotrack system determined the lowest frequency in a periodic waveform of USV at every 1 ms interval and derived fundamental frequencies. Fundamental frequencies of USVs between <30 and ≥70 kHz at the start and end points, respectively, initiated the re-analyses of USV data with the manual selection mode due to the possibility of artifacts. USVs that satisfied all of the following criteria were selected for statistical analyses [44].

i. The fundamental frequencies at both start and end points were ≥30 and <70 kHz.
ii. The duration was ≥20 ms.
iii. The mean fundamental frequency was <90 kHz.
iv. The bandwidth between the maximum and minimum fundamental frequencies was <60 kHz.

Because vocalization-related organs of the pups are immature, they cannot produce the USVs with wider bandwidth and longer durations as juvenile and adult rats can. Acoustic parameters of selected USVs considered for statistical analyses included the number, duration, fundamental frequency, bandwidth, and amplitude. Duration, amplitude, and fundamental frequencies were determined through the average USVs of PNDs 4, 8, and 12 for 10 pups in each of the three groups. Bandwidth was the range between the maximum and minimum fundamental frequencies of USVs. Moreover, the percentage of USVs with frequency modulations was calculated as (total number of USVs with frequency modulations/total number of USVs) × 100. Frequency modulation was defined as the USV with a bandwidth ≥5 kHz [64]. All USVs, including the frequency modulated ones, were the 40 kHz distress-type calls emitted by pups ranging from 40 to 55 kHz.

The acoustic parameters of the USVs were analyzed using a three-way analysis of variance (ANOVA) defined by two between-group factors, concentration (high/low/control) and sex (male/female), and one within-subject factor, age (PNDs 4/8/12/16). USV data reported in the figures were the average USVs produced by 10 pups (5 males and 5 females) in each ethanol group. On PND 16, no other acoustic parameters except the number of USVs were possible to be analyzed with ANOVA, because no USVs were produced by many pups regardless of ethanol exposure. The body weights of pups were analyzed via three-way ANOVA comprising two between-group factors of concentration and sex and one within-group factor of age. Ethanol consumption in dams was subjected to a one-way ANOVA comprising one between-group factor of concentration. When a factor was found to be significant, multiple comparisons were performed using Ryan’s method. Statistical analyses were conducted through ANOVA 4 on the website (http://www.hju.ac.jp/_kiriki/anova4/about.html, accessed on 17 June 2021).

## 3. Results

### 3.1. Daily Consumption of Ethanol-Containing Water

**Figure 1:**
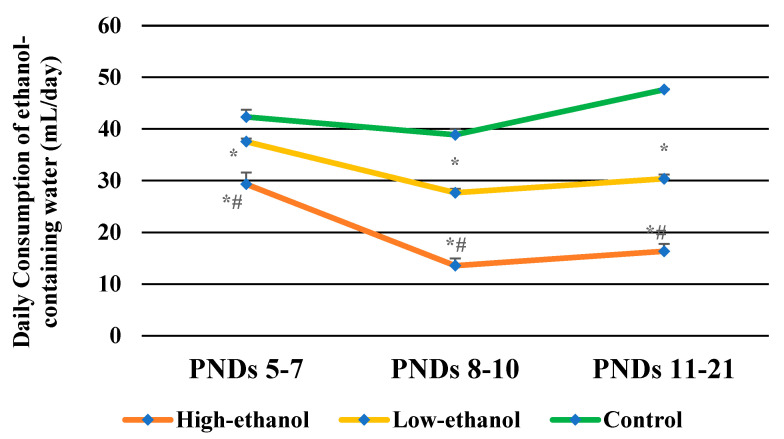
Daily consumption of ethanol-containing water in rat dams on PNDs 5–7, 8–10 and 11–21; Data are expressed as the mean ± SEM; * *p* < 0.01 compared with that in the control dams; # *p* < 0.001 compared with that in the low-ethanol dams; the number of dams was 6 in each group.

The daily intake of ethanol-containing water significantly varied across the groups (*F* (2, 15) = 177.508, *p* < 0.001). As shown in Figure 1, the daily intake of ethanol-containing water for the high-ethanol dams on PNDs 5–7 (10% ethanol), PNDs 8–10 (20% ethanol), and PNDs 11–21 (30% ethanol) was 29.33, 13.56, and 16.33 mL/day, respectively. For the low-ethanol dams, the daily consumption on PNDs 5–7 (5% ethanol), 8–10 (10% ethanol), and 11–21(15% ethanol) was 37.56, 27.67, and 30.36 mL/day, respectively. The daily consumption of pure tap water for the control dams on PNDs 5–7, 8–10, and 11–21 was 42.34, 38.89, and 47.62 mL/day, respectively. For high-ethanol dams, the daily consumption of ethanol-containing water was consistent between PNDs 8–10 and 11–21 but was significantly lower than the daily consumption of PNDs 5–7 (*p* < 0.001). Thus, it is evident that high-ethanol dams drank significantly less water with higher content of ethanol.

### 3.2. Landmarks of Physical Growth

Lactational ethanol exposure adversely affected the physical growth of pups from the group with the higher content of ethanol. Almost all landmarks of physical growth were delayed in the high-ethanol pups. The time of incisor eruption, onset of body hair, and eye openings were evidently delayed compared to those of the control pups (Table 1). More importantly, 16 of the high-ethanol pups died before weaning, whereas none of the low-ethanol and control pups were found dead. The low-ethanol and the control pups were timely weaned on PND 22, but the high-ethanol pups were weaned on PND 30 because growth retardation threatened their survival.

### 3.3. Body Weights of Dams

**Figure 2:**
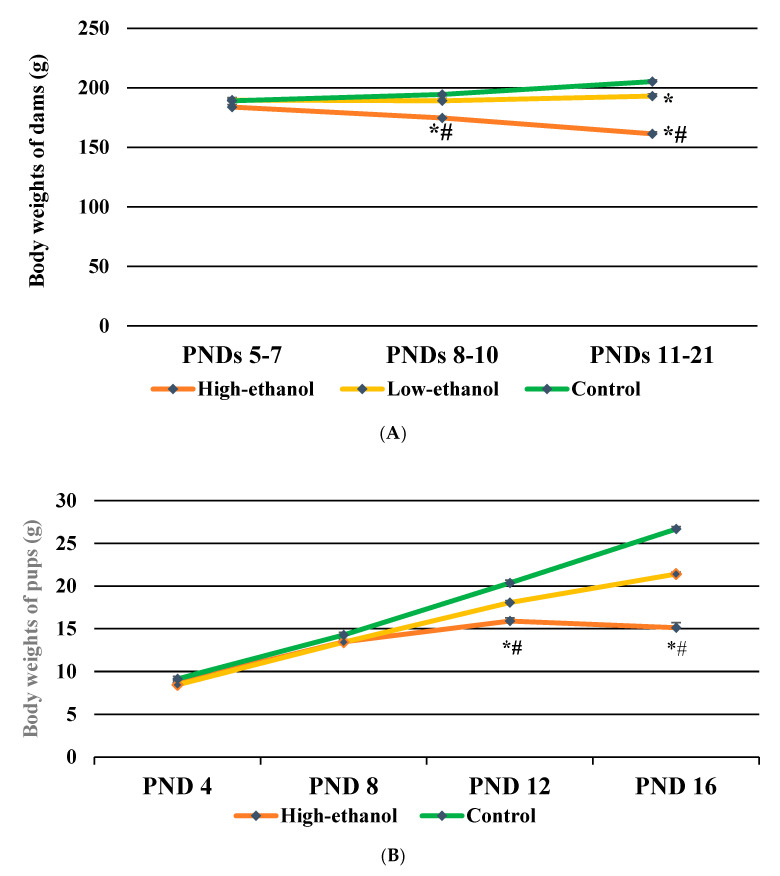
(**A**) Effects of ethanol exposure during lactation on body weights of dams; (**B**) Effects of ethanol exposure during lactation on body weights of rat pups; Data are expressed as the mean ± SEM; * *p* < 0.05 compared with that in the control dams and control pups, # *p* < 0.05 compared with that in the low-ethanol dams and low-ethanol pups; the number of dams was 6 in each of the three groups; the number of pups was 10 (5 males and 5 females) in each of the three groups.

The body weights of dams significantly varied across the groups (*F* (2, 15) = 66.912, *p* < 0.001). Dams significantly lost body weights as the concentrations of ethanol increased, exhibiting lowest body weights in the high-ethanol group followed by the low-ethanol and the control groups (*p* < 0.05). A two-way interaction between ethanol concentration and the timing of exposure (PNDs) was significant (*F* (4, 30) = 85.453, *p* < 0.001, Figure 2A). The average body weights of high-ethanol dams on PNDs 8–10 and 11–21 were significantly reduced compared to the low-ethanol and control dams (*p* < 0.05).

### 3.4. Body Weights of Pups

The body weights of individual pup were measured just after the USV recording on PNDs 4, 8, 12, and 16. The body weights significantly varied across the groups (*F* (2, 24) = 86.371, *p* < 0.001). Reductions in body weights were evident as the concentration of ethanol increased, showing lowest body weights in the high-ethanol pups followed by the low-ethanol and the control pups (*p* < 0.05). A two-way interaction effect between ethanol and age was significant (see Figure 2B) (*F* (6, 72) = 131.024, *p* < 0.001). On PNDs 12 and 16, the high-ethanol pups significantly lost their body weights compared to the low-ethanol and control pups (*p* < 0.05). An age-dependent elevation in body weights was significant in all three ethanol groups (*F* (3, 72) = 1670.920, *p* < 0.001). Sex differences in body weights of the pups were not significant (data not shown).

### 3.5. Number of USVs

**Figure 3:**
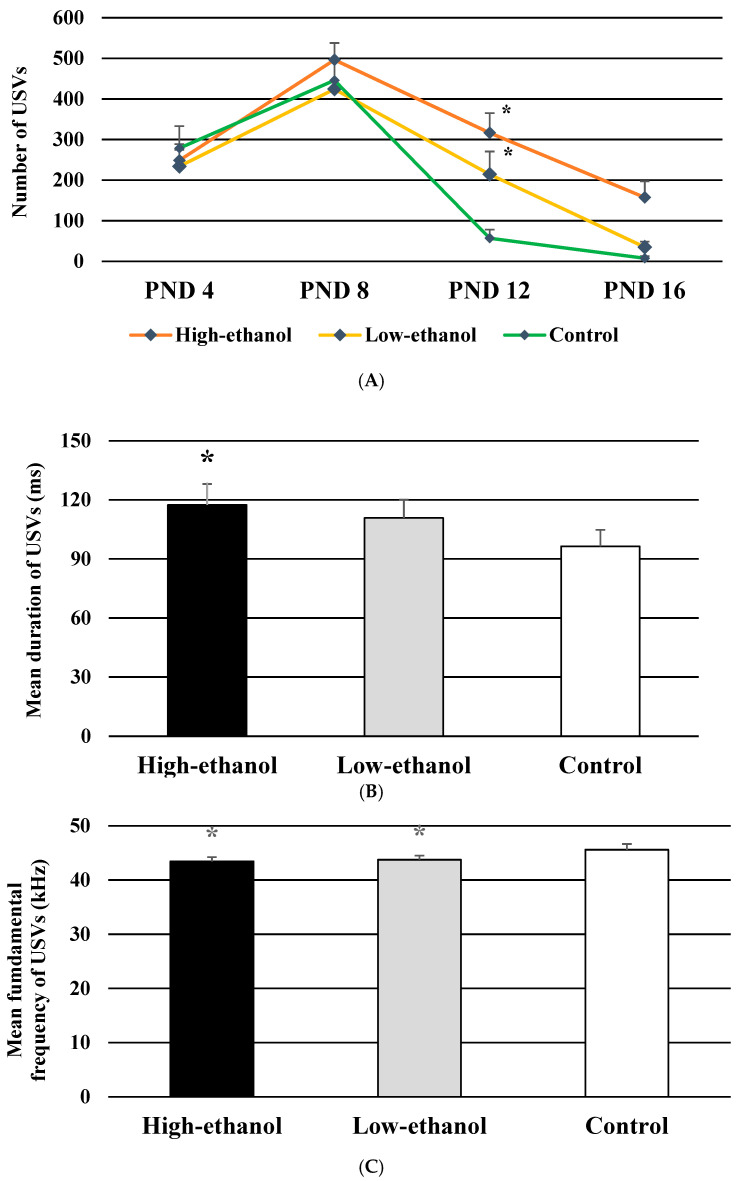

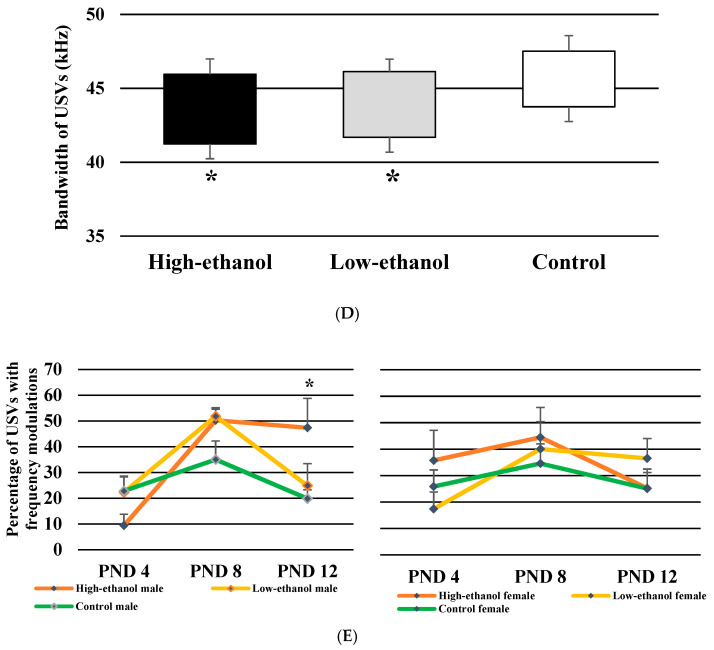
(**A**) Effects of ethanol exposure during lactation on the number of USVs in rat pups; data are expressed as the mean ± SEM; * *p* < 0.05 compared with that in the control pups; the number of pups was 10 (5 males and 5 females) in each of the three groups. (**B**) Effects of ethanol-exposure during lactation on the mean duration of USVs in rat pups; data are expressed as the mean ± SEM; * *p* < 0.05 compared with that in the control pups; the number of pups was 10 (5 males and 5 females) in each of the three groups. (**C**) Effects of ethanol-exposure during lactation on the mean fundamental frequency of USVs in rat pups; data are expressed as the mean ± SEM; * *p* < 0.05 compared with that in the control pups; the number of pups was 10 (5 males and 5 females) in each of the three groups. (**D**) Effects of ethanol-exposure during lactation on the bandwidth of USVs in rat pups; the lower and upper segments of bandwidth indicate the minimum and maximum fundamental frequencies, respectively; data are expressed as the mean ± SEM; * *p* < 0.05 compared with that in the control pups; the number of pups was 10 (5 males and 5 females) in each of the three groups. (**E**) Effects of ethanol-exposure during lactation on the percentage of USVs with frequency modulations in rat pups; data are the mean ± SEM; * *p* < 0.05 compared with that in the control male pups; the number of pups was 5 in each of the six groups.

An elevated number of USVs was evident by the main effects of ethanol (*F* (2, 24) = 4.789, *p* < 0.05). Production of the greater number of USVs was prominent in the high-ethanol pups than the control pups (*p* < 0.05). The significant interaction between ethanol and age (*F* (6, 72) = 2.318, *p* < 0.05, Figure 3A) reflected the elevations of USVs on PND 12 in both the high- and low-ethanol pups compared to the control pups (*p* < 0.05). The main effect of age on the number of calls (*F* (3, 72) = 42.041, *p* < 0.001) exhibited that the highest number of USVs was produced on PND 8 followed by those on PNDs 4, 12, and 16 (*p* < 0.05). Sex effects on the number of emitted USVs were not significant (data not shown).

### 3.6. Mean Duration of USVs

The duration of USVs significantly varied across the groups (*F* (2, 24) = 3.494, *p* < 0.05, Figure 3B). Longer calls were produced by pups from the high-ethanol group than in the control group (*p* < 0.05). Sex of the pups had no significant effects on the mean duration of USVs.

### 3.7. Mean Fundamental Frequency of USVs

Ethanol had significant group effects on the fundamental frequency of USVs (*F* (2, 24) = 4.226, *p* < 0.05, Figure 3C). Lower fundamental frequencies were produced by the high- and low-ethanol pups than the control pups (*p* < 0.05). Sex differences in the mean fundamental frequency of pup USVs were not significant.

### 3.8. Bandwidth of USVs

Ethanol exposure had no significant effects on the bandwidth of USVs. However, significant effects were evident in minimum fundamental frequencies of USVs (*F* (2, 24) = 7.625, *p* < 0.005, Figure 3D). Decreased minimum fundamental frequencies were noticed in the high- and low-ethanol pups compared to the control pups (*p* < 0.05). Minimum fundamental frequencies of USVs significantly varied across the age of the pups (*F* (2, 48) = 6.203, *p* < 0.005). With the advancement of age, the minimum fundamental frequencies were decreased on PNDs 8 and 12 compared with those on PND 4 (*p* < 0.05). Maximum fundamental frequencies of USVs were not affected by ethanol exposure. Sex had no significant effects on USV bandwidth as well as on the minimum and maximum fundamental frequencies of USVs.

### 3.9. Percentage of USVs with Frequency Modulations

A three-way interaction of ethanol, sex, and age on the percentage of USVs with frequency modulations was significant (*F* (4, 48) = 3.624, *p* < 0.05, Figure 3E), exhibiting that the high-ethanol male pups produced greater percentage of USVs with frequency modulations than the control male pups on PND 12 (*p* < 0.05). A significant age effect was observed (*F* (2, 48) = 15.453, *p* < 0.001) as reflected through elevations in the percentage of frequency modulated USVs on PND 8 compared with those on PNDs 4 and 12 (*p* < 0.05).

### 3.10. Amplitude of USVs

The amplitude of USVs had no significant main effect or interaction effect of ethanol and sex. However, the age effects were significant (*F* (2, 48) = 7.079, *p* < 0.005) indicating greater amplitudes on PND 8 than those on PNDs 4 and 12 (*p* < 0.05) (data not shown).

In summary, these findings suggest that the gradually elevated exposure of ethanol concentrations in the high-ethanol dams on PNDs 5–7, 8–10 and 11–21 resulted in lower daily consumption of ethanol-containing water with higher content of ethanol. Accordingly, a significant loss in body weight of both the high-ethanol dams and their pups was noticed and USV acoustic parameters upon pups’ isolation from the dams were altered, showing elevations in pups’ distress calls with longer duration and higher percentage of frequency modulations.

## 4. Discussion

Ethanol consumed by rat dams is secreted into breast milk and readily transmitted into pups, exerting direct impacts on them. For dams, ethanol exposure during lactation reduced the breast milk production [17,48] and induced poor maternal care in building nest, retrieving pups, and crouching for breast feeding [65,66]. The pups from those ethanol intoxicated dams exhibited suckling deficits [19] and, in turn, suffered from malnutrition contributing to retarded growth [67,68]. Growth retardation due to ethanol exposure (2.5 g/kg/day on PNDs 3–13) affected the motor coordination in pups necessary for nipple attachment, which was reflected through decreased perioral responsiveness and longer latencies to attach to the nipples [69,70]. Growth retardation might also affect the vocalization related organs of ethanol-exposed pups during lactation and alter their communication with dams.

### 4.1. Physical Growth

The pups of the high-ethanol group in the present study carried the same body weights on PNDs 12–16 indicating growth retardation, whereas the body weights of control pups were steadily increasing on each PND. Other physical landmarks like body hair growth, incisor eruptions, and eye-openings were also delayed in high-ethanol pups compared with the low-ethanol and control pups. Due to growth retardation, weaning of the high-ethanol pups was delayed up to PND 30. In the present study, consumption of 30% ethanol-containing water resulted in lower intake (16.33 mL/day) by the high-ethanol dams, whereas consumption of 15% ethanol-containing water resulted in higher intake (30.36 mL/day) by the low-ethanol dams from PNDs 11 to 21. Growth retardation was highly evident in high-ethanol pups, whereas low-ethanol pups had no such evidence. Therefore, higher ethanol-containing water (30%) with lower intake (16.33 mL/day) was more detrimental than lower ethanol-containing water (15%) with greater intake (30.36 mL/day).

The parameters of physical growth of high-ethanol pups in the present study were found to be highly consistent with the results of earlier studies [19], where lactational ethanol exposure at a dose of 6 g/kg/day on PNDs 4–12 resulted in retarded body weights in pups compared to those of other treatment groups. Moreover, delayed eye openings and incisor eruptions altered the sensory modalities related to nipple attachment and contributed to the suckling deficits in ethanol-exposed (6 g/kg/day) pups. Combined gestational and lactational exposure to 20% ethanol concentrations resulted in lower intake of ethanol in dams and reductions in the body weights of pups [47]. Lower intake of higher ethanol-containing water (20%, *v*/*v*) made the dams dehydrated, reflected through deficiencies in maternal nutritional status and milk production. Consequently, sucking pups of high-ethanol exposed dams were malnourished and exhibited decreased body weights [18] similar to those of other studies with higher ethanol concentrations (25% ethanol) [48]. Repeated states of ethanol intoxication (1 and 2 g/kg/day) either in the early (PNDs 5–8) or mid-stage (PNDs 9–12) of lactation resulted in decreased breast milk production in rat dams and growth retardation in the pups [71,72]. In addition, ethanol (2.5 g/kg/day) administered to dams on PNDs 3–13 impaired the capability of the dams to retrieve the pups and disrupted the fixed action patterns relevant to maternal care [50]. Insufficient maternal care, in turn, led to lower body weights, increased mortality rates, and other growth retardation in pups observed in the present study. All these results suggest that the effects of ethanol on pups could be caused by its effects on the dams. Exposure to higher ethanol-containing water caused lower water consumption, resulted in decreased body weights, and presumably dehydration and less milk produced in the dams. Resulting inappropriate maternal care to pups was evident, so they were unsuccessful in sucking behaviors and displayed growth retardation and malnutrition up to the point of death.

### 4.2. USV Acoustic Parameters

The main purpose of the study was to determine the acoustic parameters of USVs of rat pups upon their isolation from the dam, because few studies have been carried out on the dam–pup interactions in the USV domain and comparing them with results of behavioral studies. In our study, increased numbers of USVs with longer duration and frequency modulations were prominent in high-ethanol pups compared with the control pups. These USV alterations were particularly evident on PND 12 along with the inhibition of body weight gains in the high-ethanol pups. Consistent with this is the study in [20], where intragastric ethanol administration on PNDs 3, 5, 7, 9, 11, and 13 at doses of 2.5 g/kg/day to rat dams resulted in increased USVs in pups on PNDs 3 and 9, as indexed by behavioral distress. Conversely, in [45,73] researchers administered ethanol (6 g/kg/day) intragastrically to rat pups on PNDs 4–10 and PNDs 1–7. The rat pups in both studies had longer latencies to produce the first vocalization, and produced decreased number of USVs. Similarly, intragastric ethanol administration (3 g/kg/day) to rat pups on PNDs 4–10 resulted in lower number of USVs on PND 14 [30]. However, intragastric administration of ethanol could have direct adverse effects on the organs and tissues of the vocal tract and larynx, and potentially affect production of USV. From the findings of the present study, it is reasonable to argue that the decreased body weights of the high-ethanol dams were caused by dehydration, and consequently, lowered amount of milk production and altered maternal care of their stressed pups.

Regardless of the effects of pup undernutrition following disrupted maternal care, significant elevations in the number of distress calls in both the high- and low-ethanol pups compared to the control pups could be because of ethanol itself. Importantly, the body weights of low-ethanol dams and their pups did not differ with those of the controls in the present study. Other acoustic parameters of USVs except for the number of distress calls created no differences between the low-ethanol and control pups. Thus, lower ethanol-containing water with greater intake in low-ethanol dams did not cause dramatic undernutrition, and promoted sufficient maternal care to their pups.

### 4.3. Comparisons between Gestational and Lactational Exposure

The effects of gestational ethanol exposure on USVs in rat pups have been demonstrated in earlier relevant studies [41,42,63]. These studies reported that prenatal exposure to ethanol reduces the number of USVs [41,63], except in one study [42] where perinatal exposure increased the number of USVs in pups. However, the pattern, dose, and duration of ethanol exposure as well as the acoustic parameters of USVs investigated in these studies varied grossly or seemed insufficient to draw a valid conclusion. Towards this end, in our recent research [44], ethanol was administered to pregnant rats on gestational days 8–20 via drinking water. On GDs 8–10, 10% and 5%, on GDs 11–13, 20% and 10%, and on GDs 14–20, 30% and 15% ethanol-containing water (*v*/*v*) was administered to the rats of the high- and low-ethanol groups, respectively. A higher number of distress USVs, elevated fundamental frequencies, and higher amplitudes were displayed by the high-ethanol pups (30%) compared to both the low-ethanol (15%) and control (0%) pups. Distress USVs emitted by high-ethanol pups indicated their negative emotionality upon maternal isolation [44]. However, pups in all three groups underwent similar body weight gains from infancy to adulthood, and other growth indices such as the time of ear and eye openings, body hair growth, and incisor eruptions, were not delayed in gestational exposure. They were weaned on PND 21 regardless of ethanol administration and the survival rate of pups in all three groups was 100% [44]. In gestational exposure, ethanol intake by pregnant dams is readily transmitted into the blood circulation and crosses the placental barrier to reach into the fetuses. However, gestational period showed limited effects on maternal behavior. After parturition, the dams were not exposed to ethanol anymore and they were able to ensure proper maternal behavior. Consequently, the pups from all ethanol groups grew up normally and weaned on PND 21 [44].

Contrary to the effects of gestational exposure, lactational high-ethanol exposure in the present study resulted in growth retardation, as reflected through delayed eye openings and incisor eruptions in pups. In addition to that, pups also showed signs of malnutrition. Therefore, it is likely that the high-ethanol pups were not able to receive sufficient breast milk from the dams and suffered from malnutrition leading to growth retardation. Consistent with this is the observation that the high-ethanol dams were unable to ensure proper maternal behavior such as retrieving the pups and adopting a crouching posture due to ethanol intoxication. Accordingly, pups of the high-ethanol group sought more maternal care and produced greater number of USVs demonstrating distressful emotional states. Disruptions in maternal care after ethanol consumption during lactation included the incapability of ethanol-consumed dams to retrieve the pups, to facilitate licking or grooming, and to adopt a crouching posture as revealed in previous studies [49,51,66]. It was also shown that time spent with the pups was reduced and time required for building nest was increased in ethanol-exposed dams and litter fragmentation was elevated [74]. Previous research [75] revealed that pups produce more distress USVs when they are isolated from the nest and number of USVs reached its peak when the pups were alone. Being isolated from the dam in the present study, pups of the high-ethanol group produced greater number of USVs but with longer duration and frequency modulations, indicating elevated negative emotionality. Adverse effects of lactational ethanol exposure on pups has been revealed in past studies as well. Researchers [19,69] found longer latencies for ethanol-exposed pups to attach to nipples, less time spent in suckling, and retarded body weight gains.

We randomly selected 10 pups from the 48 high-ethanol pups and used them for USV recording. All these 10 pups survived and weaned. However, 16 out of the remaining 38 high-ethanol pups died before weaning. Previous studies [54,74] reported that distress calls produced by pups serve as a biological signal leading to arousal and caregiving behavior of the dams and importantly, that rat dams gave more maternal care to isolated pups compared to pups staying in the nest all the time, and spent more time with the isolated pups and frequently showed licking and grooming behaviors. All acoustic parameters contribute to localize the pups by the dam [76] and facilitate appropriate maternal care, hence in the present study, all crucial acoustic parameters of USVs were examined. The high-ethanol pups in the present study produced distress USVs with frequency modulations upon maternal isolation, and continuation of USV emission after reunion with ethanol-intoxicated dam provoked arousal in the dam to facilitate maternal care, although it appeared insufficient for many endangered pups. Altogether, it is well-evident that lactational high-ethanol exposure has more serious consequences than the gestational one and induces more harmful effects in pups.

### 4.4. Central Mechanisms of Ethanol Exposure and Negative Emotionality

The tegmental structures in the midbrain are considered as the direct initiators of USVs in rats [77,78,79]. Negative emotionality is closely related to the enhanced neuronal activity in laterodorsal tegmental nucleus (LDT) [80] but not in pups [81]. LDT neurons play a functional role in negative emotion-induced USVs upon maternal isolation [82]. LDT is considered as one of the sources of GABAergic input to the ventral tegmental area (VTA) [83]. GABAergic innervation to the VTA originates from the rostromedial tegmental nucleus (RMTg) along with other brain regions. As an essential inhibitory afferent to midbrain dopamine neurons, the RMTg is activated during ethanol exposure [84,85,86] and the inhibitory RMTg GABA input to VTA dopaminergic (DA) neurons plays a role in regulating negative emotionality [87]. In the present study, the high-ethanol pups exhibited USV acoustic alterations reflected through a greater number of distress USVs with longer duration and a higher percentage of frequency modulations. Acoustic alterations in USVs indicate prominent elevations of negative emotionality and this might be due to the ethanol-induced excitation of RMTg neurons exhibited through the elevated distress calls in pups. However, the expression of the RMTg GABA input to the VTA after ethanol exposure and the resulting distressful states are different in adult rats compared to rat pups [88,89] and thus, further relevant studies on rat pups will be required to settle this issue.

In conclusion, all these results suggest that ethanol-induced distressful emotional states in pups might be because of ethanol effects on dams and/or due to the central nervous system effects of ethanol on pups. In the present study, elevations in the number of distress USVs in the high- and low-ethanol pups could be because of the ethanol-induced activation of RMTg that transmitted inhibitory GABA input to VTA DA neurons to exhibit negative emotionality in pups. Another explanation is that lower water consumption with higher ethanol content resulted in undernutrition and less milk produced in the high-ethanol dams. Accordingly, their pups were malnourished and exhibited unsuccessful suckling behaviors. Resulting malnutrition and growth retardation in the high-ethanol pups forced them to produce more distress calls with longer durations and frequency modulations upon isolation from the dams, as indicative of elevated negative emotionality.

## Figures and Tables

**Table 1:** Effects of lactational ethanol exposure on landmarks of physical growth in rat pups.

Pups	Incisor Eruption (PNDs)	Body Hair (PNDs)	Eye Opening (PNDs)	Timing of Death (PND)	Weaning
High-ethanol	15.094 ± 0.222	11.844 ± 0.191	20 ± 0.174	18 ± 0.224 (n = 16)	PND 30
Low-ethanol	11 ± 0.179	8.75 ± 0.113	16.708 ± 0.168	No death	PND 22
Control	9.271 ± 0.142	8.167 ± 0.124	15 ± 0.139	No death	PND 22

Note: The sample size for the high-ethanol, low-ethanol and control pups is 32, 48, and 48, respectively. Physical landmarks of sixteen dead pups in the high-ethanol group have not been incorporated in Table 1. Data are expressed as the mean ± SEM.
